# Effect of Fluid Shear Stress on Portal Vein Remodeling in a Rat Model of Portal Hypertension

**DOI:** 10.1155/2015/545018

**Published:** 2015-03-29

**Authors:** Bin Wen, Jian Liang, Xin Deng, Ran Chen, Peichun Peng

**Affiliations:** Ruikang Hospital, Guangxi Chinese Medicine University, Nanning, Guangxi 530011, China

## Abstract

*Aims*. To explore the effects and mechanisms of fluid shear stress on portal vein remodeling in a rat model of portal hypertension. *Methods*. Subcutaneous injections of CCl4 were given to establish a rat model of liver cirrhosis and portal hypertension. Biomechanical technology was adopted to determine the dynamic changes of haemodynamic indices and fluid shear stress. Nitric oxide (NO), synthase (NOS), and endothelin-1 (ET-1) of the portal vein blood were measured. Changes in geometric structure and ultrastructure of the portal vein were observed using optical and electron microscopy. *Results*. After the CC14 injections, rat haemodynamics were notably altered. From week 4 onwards, PVP, PVF, and PVR gradually and significantly increased (*P* < 0.05 versus baseline). The fluid shear stress declined from week 4 onwards (*P* < 0.01 versus control group). NO, NOS, and ET-1 increased after repeated CCI4 injections. Hematoxylin and eosin staining showed thickened portal vein walls, with increased inside and outside diameters. Electron microscopy revealed different degrees of endothelial cell degeneration, destruction of basement membrane integrity, proliferating, and hypertrophic smooth muscle cells. *Conclusions*. Fluid shear stress not only influenced the biomechanical environment of the portal vein but also participated in vascular remodeling.

## 1. Introduction

Portal hypertension results from elevations in both portal resistance and blood flow. Hemodynamic alterations are not only manifestations of vascular remodeling but also a key factor for maintaining portal hypertension [1]. Vascular remodeling refers to environmental changes leading to structural and functional alteration of blood vessels [2]. This is mainly characterized by abnormal blood vessel gene expression and the migration, hyperplasia, hypertrophy, and apoptosis of the endothelial cells (ECs) and smooth muscle cells (SMCs). Stress factors acting on the blood vessels result in structural and functional remodeling of the arterial walls. In particular, the significant biomechanical effect of shear stress on ECs has been widely acknowledged [3]. A number of studies have reported that shear stress is associated with vascular remodeling [4, 5]. Shear stress can promote remodeling of the arterial walls, possibly through regulation of EC gene expression and intracellular bioactive substances [6, 7].

Over recent years, abnormal venous wall remodeling regulated by stress has become a topic of intense interest. Longitudinal stretching stress leads to human venous remodeling in terms of both structure and function [8, 9]. Venous remodeling is mainly associated with the proliferation of SMCs [10, 11]. However, it is generally thought that abnormal venous wall remodeling is closely related to shear stress, since the intima is directly exposed to this stress. Until recently, there has been little understanding of the relationship between shear stress and vascular remodeling. Therefore, further research on the mechanisms of portal remodeling based on shear stress is warranted.

In this study, the changes in vasoactive factors secreted by the portal vein and the remodeling of vascular walls were investigated under conditions of shear stress. This study aimed to further investigate portal venous remodeling in the pathogenesis of liver cirrhosis with portal hypertension, thereby guiding its prevention and treatment.

## 2. Materials and Methods

### 2.1. Animals and Instruments

Specific pathogen-free (SPF) male laboratory Sprague-Dawley rats, aged 6 to 7 weeks and weighing 180 ± 20 g, were provided by the Animal Laboratory Center of Guangxi Medical University (Nanning, China; quality certificate number: SCXK2009-0002). Nitric oxide (NO), endothelin-1 (ET-1), and NO synthase (NOS) test kits (Shanghai BoyaoBiological Engineering Co., Ltd.), an MFV-3200 electromagnetic flow meter, an RM-6300 eight-channel physiological record instrument (Nihon Kohden, Japan), a pressure transducer, an SMUP-PC biological signal processing system, Image Measurement 1.00 software, and the MFLab2.00 software package were all provided by the Department of Physiology at the Guangxi University of Traditional Chinese Medicine, Nanning, China. An H-7650 transmission electron microscope (Hitachi Ltd., Japan) was also available through the department.

### 2.2. Testing Indexes and Methods

#### 2.2.1. Establishment of Rat Liver Cirrhosis by Portal Hypertension

After adaptive feeding for one week, the rats were randomized into the CCl4 group (*n* = 42) and the control group (*n* = 8). The CCl4 group received subcutaneous injections of CCl4 (40% in Betis olive oil, 0.5 mL/100 g) initially, given every three days (0.3 mL/100 g). The animals were fed 15% alcohol in water as the only drink and as food a mixture of lard (20%), cholesterol (0.5%), and corn powder (79.5%) [12]. The control group was fed with ordinary chow and pure water. Eight rats were taken out from the CCl4 group at weeks 4, 6, 8, and 10, respectively, for monitoring of indices and pathological analysis of the liver tissues.

#### 2.2.2. Determination of Portal Haemodynamics

After fasting for eight hours before the determination of blood flow and portal pressure, rats were anesthetized with an injection of 10% chloral hydrate (3 mL/kg) in the abdominal cavity. After routine disinfection of the skin, a subxiphoid median incision was made and the surface tissues were peeled off layer by layer to dissociate the main portal vein. An appropriate electromagnetic probe was inserted into one side, and portal vein flow (PVF) was measured with an MFV-3200 electromagnetic flow meter. The mean value was recorded after the blood flow was stable, and then the mesenteric vein was dissociated. A PV-50 catheter was inserted into the main portal vein. Portal venous pressure (PVP) was determined using the pressure transducer and eight-channel physiological record instrument. All operations were conducted at a temperature of 25°C. Heparin sodium-saline solution was used to wash the pipes before determination. Data were recorded, entered into a computer, and subsequently processed using the MFLab2.00 software package. The portal vein resistance (PVR) was calculated based on the formula PVR = PVP/PVF.

#### 2.2.3. NO, ET-1, and NOS Detection in the Portal Vein Blood

Blood was drawn from the portal vein in tubes with anticoagulant and centrifuged for 10 minutes to collect the plasma. The kits were strictly used according to the manufacturer's instructions. The nitrate reductase method was adopted to calculate NO plasma concentrations. NOS activity was measured with a chemical colorimetric method. Radioimmunoassay was applied to determine the content of ET-1 in plasma.

#### 2.2.4. Calculation of the Shear Stress

Using the measured PVP, PVF, and PVD, dynamic changes of the shear stress in portal veins were calculated based on the formula *T* = 4*μQ*/*πr* ^3^, in which *Q* stands for PVF, *μ* for blood viscosity, and *r* for PVD/2 [13].

#### 2.2.5. Geometric Structures under the Optical Microscope

The main portal vein (middle segment) was fixed in a 10% formaldehyde solution. After routine hematoxylin and eosin (HE) staining, the values of the inside diameter (ID), outside diameter (OD), wall thickness (WT), and vascular cross-sectional area (CSA) were observed under an optical microscope and were measured using a computer image analysis system.

#### 2.2.6. Ultrastructure of the Portal Vein by Electron Microscopy

Fresh main portal veins from the same sites were dissected into sections (1 mm × 1 mm × 1 mm), which were then put into 4% glutaraldehyde precooled at 4°C, fixed for 24 hours, and then fixed in 1% osmium tetroxide for 2 hours. Then ethyl hydroxide and dimethyl ketone were used for dehydration. Acetone-EPON812 epoxide resin was added for embedding, trimming, and slicing. After staining with uranyl acetate and lead citrate, ECs and SMCs in the portal vein were observed under the transmission electron microscope.

### 2.3. Statistical Analysis

The data were expressed as mean ± standard deviation, and SPSS 17.0 software was used to carry out independent sample* t*-tests and analysis of variance. *P* values <0.05 indicated significant differences.

## 3. Results

### 3.1. General Condition of the Rats

The rats in the control group were in good health during the experiment, with lustrous fur, good appetites, and increase in body weight. All control rats survived during the experiments. Four weeks after injection of CCl4, treated rats appeared less healthy, with body weight loss, poor appetite, unhealthy fur, and yellow urine. Ten treated rats died during the experiment.

### 3.2. Pathological Changes of the Liver Tissues

**Figure 1:**
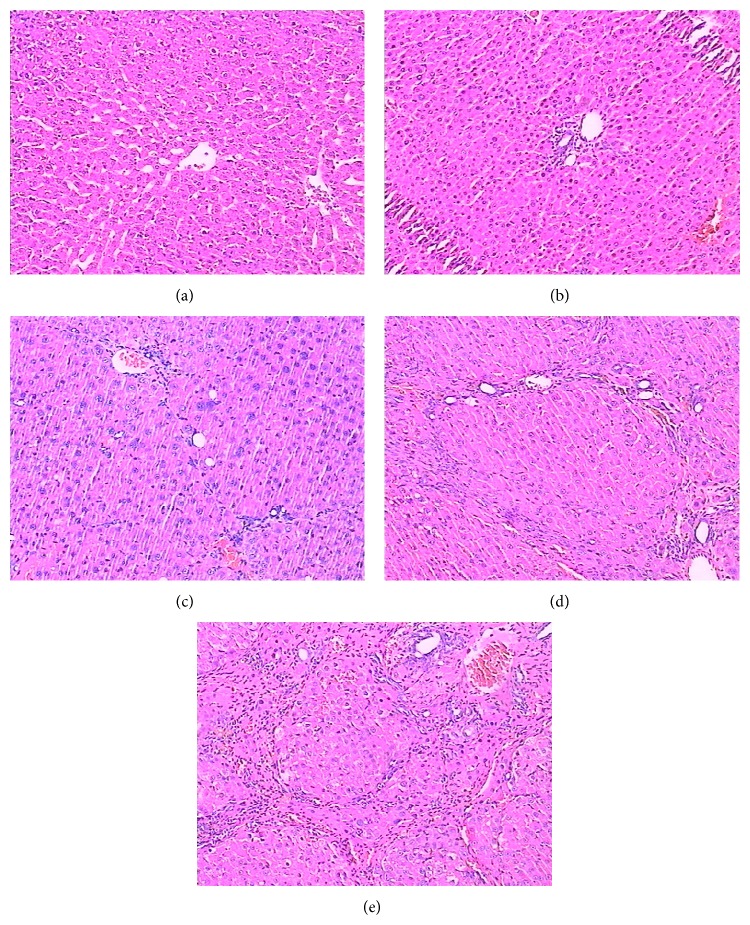
Hematoxylin and eosin staining of the liver tissues. (a) Control group, (b) week 4, (c) week 6, (d) week 8, and (e) week 10 after CCl4 injection.

HE staining showed structurally integrated liver tissues, with a clear lobular structure and regularly arranged hepatocytes in a cord-like shape (Figure 1(a)). Four weeks after the initial CCl4 injection, the pathological changes in the rat of the 4-week model showed hepatic fat degeneration, cellular swelling, and necrosis of hepatocytes (Figure 1(b)). After six weeks, inflammatory cell infiltration of the liver tissues had increased, and fibril connective tissue of the portal area showed proliferation (Figure 1(c)). At eight and ten weeks after injection collagen deposits were observed in the lobules and the formation of hepatic pseudolobules was widely present, with fibrous septa and obvious hepatocellular regenerated nodules (Figures 1(d) and 1(e)).

### 3.3. Dynamic Changes in the Haemodynamic Indexes

In the control group of rats, the average PVF, PVP, and PVR were 48.73 ± 2.38 mL/min^−1^/kg^−1^, 4.88 ± 0.29 mmHg, and 0.090 ± 0.005 mmHg/mL/min^−1^/kg^−1^, respectively. At week 4, PVF had significantly increased reaching its peak at week 8 (*P* < 0.01 versus control group), but the difference in the PVF between weeks 6 and 10 was not significant (*P* > 0.05). From week 4 onwards, PVP and PVR gradually and significantly increased (*P* < 0.05 versus baseline) and were significantly different between rats with portal hypertension and control rats after week 6 (*P* < 0.01). These results indicated successful establishment of portal hypertension in this rat model (Table 1).

### 3.4. Dynamic Changes in Shear Stress

**Figure 2:**
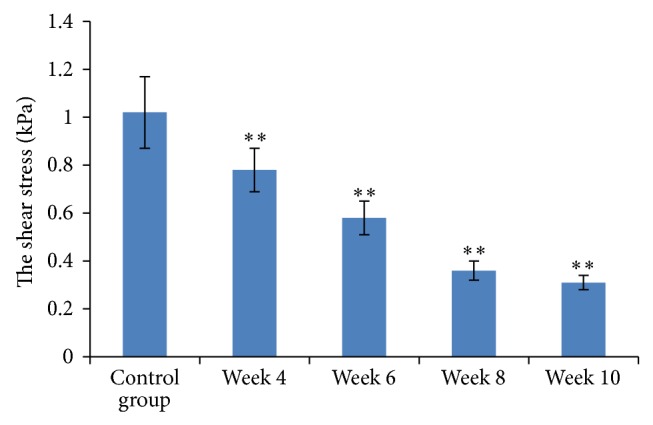
Shear stress measurements and comparison between the groups. Results are expressed as means ± SD, ^∗∗^ *P* < 0.01.

According to the formula *T* = 4*μQ*/*πr* ^3^ (in which *Q* stands for PVF, *μ* for blood viscosity, and *r* for PVD/2), the shear stress was significantly reduced from week 4 onwards (Figure 2), which is different compared to the control group (*P* < 0.01).

### 3.5. Dynamic Changes in Portal Vasoactive Substances

**Figure 3:**
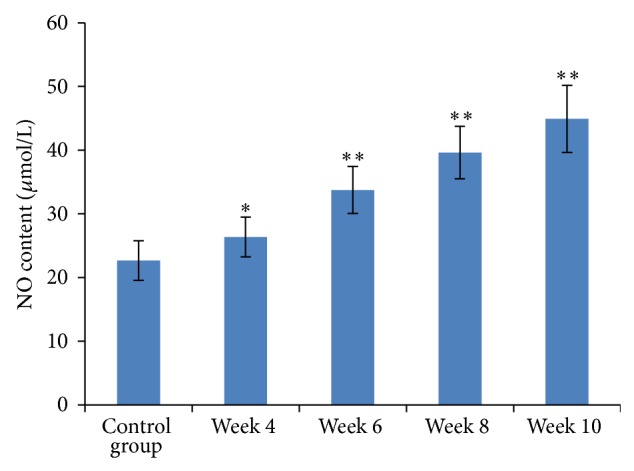
Measurement of NO content in portal venous blood. Results are expressed as means ± SD, ^∗^ *P* < 0.05 and ^∗∗^ *P* < 0.01 versus the control group.

**Figure 4:**
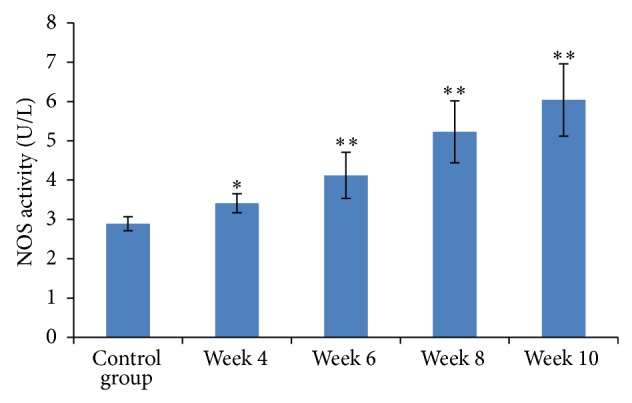
NOS activity in portal venous blood. Results are expressed as means ± SD, ^∗^ *P* < 0.05 and ^∗∗^ *P* < 0.01 versus the control group.

**Figure 5:**
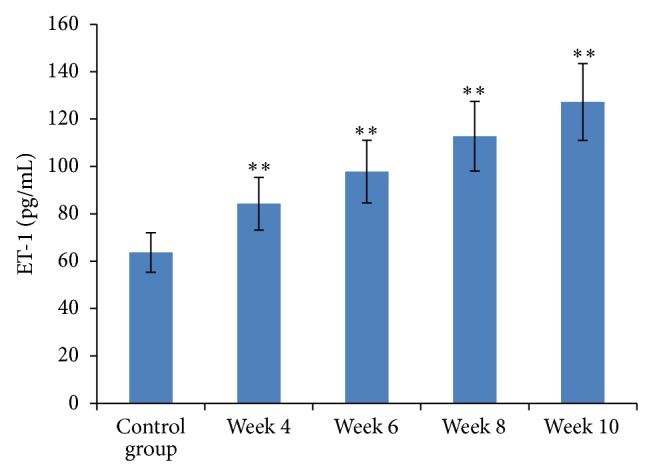
ET-1 content in portal venous blood among the groups. Results are expressed as means ± SD, ^∗∗^ *P* < 0.01 versus the control group.

From week 4 onwards, both NO and NOS in portal venous blood were significantly elevated compared to baseline levels (*P* < 0.05). They were significantly different from the beginning of week 6, as compared to the control group (*P* < 0.01, Figures 3 and 4). Plasma levels of ET-1 significantly increased four weeks after start of the CC14 injections compared to baseline levels (*P* < 0.01, Figure 5).

### 3.6. Dynamic Changes of Portal Vein Geometrical Morphology

**Figure 6:**
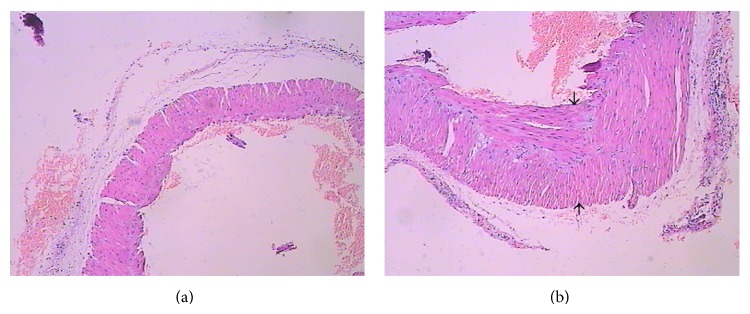
Hematoxylin and eosin staining of the portal vein. (a) Control group and (b) week 10 after CCl4 injection.

HE staining and computer image analysis at week 10 indicated thickened venous walls, widened inside diameter (ID) and outside diameter (OD), an increased wall to cavity ratio, hyperplasia of SMC hypertrophy, and nodular hyperplasia of ECs. Compared with the control group, ID, OD, wall thickness (WT), and cross-sectional area (CSA) all showed a trend towards increased values, and significant differences from the control group were observed as of week 6 after injection of CCl4 (*P* < 0.01, Figure 6 and Table 2).

### 3.7. Ultrastructural Changes of the Portal Vein

**Figure 7:**
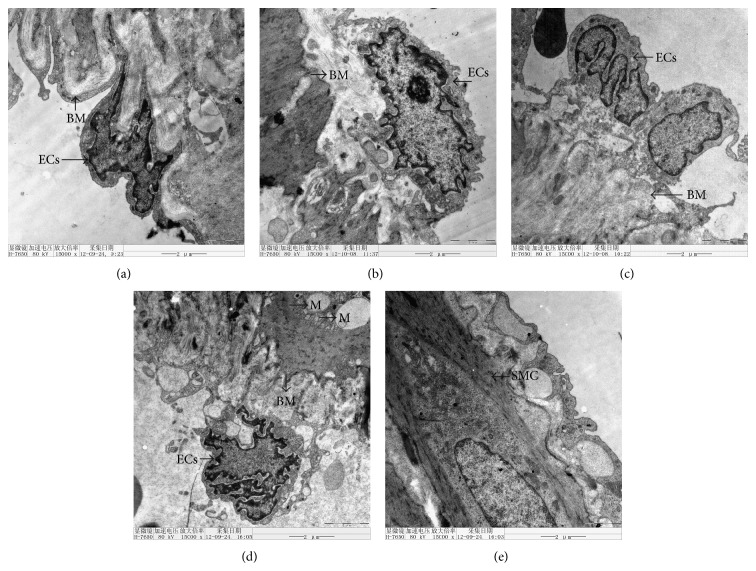
Ultrastructural changes of the portal vein after CC14 injections. (a) Control group, (b) week 4, (c) week 6, (d) week 8, and (e) week 10 after CCl4 injection. ECs, endothelial cells; SMC, smooth muscle cell; M, mitochondrion; BM, basement membrane. Magnification 15,000x.

In the control group, the structure of the ECs in the portal vein presented with intact morphology, a clear nucleus, and an intact normal basement membrane. After the injection of CCl4, abnormal ultrastructural changes of the portal vein were observed. At week 4, the portal basement membrane was thickened, but the morphology of the EC was intact and closely connected with the basement membrane without shedding. Morphology of the organelles (such as mitochondria) was rather normal. At week 6, the morphology of ECs was irregular, nucleoli were not obvious, and the basement membranes were loosely connected. At weeks 8 and 10, the integrity of basement membranes was destroyed and shedding of ECs was observed. Smooth muscle cells (SMCs) proliferated and hypertrophy was present. SMCs contained swollen and vacuolated mitochondria (Figure 7).

## 4. Discussion

The wall of the portal vein is influenced by shear stress as well as circumferential and longitudinal stress [13]. Endothelial cells (ECs), located at the lining of the blood vessels, are the cells exposed to shear stress and have the capacity to respond to blood flow changes [14]. The contribution of shear stress to vascular remodeling is mainly based on signaling by and from the ECs [6, 7]. Therefore, abnormal ECs are a key indicator for vascular remodeling.

At present, remarkable achievements have been gained in the field of cardiovascular research with regard to the effect of shear stress on vascular remodeling. Blood vessels change their structure and function under conditions of high blood pressure and low shear stress [3–5]. However, the underlying mechanism is still unclear. It is highly likely that multiple mechanisms may be associated with vascular remodeling in the portal vein. The gene expression of ECs exposed to shear stress is closely related to vascular remodeling and the progression of vasculopathy [15]. Lack of the desmuslin gene also results in phlebeurysm and leads to venous remodeling [16]. In our study we observed that, from week 4 onwards, there was a trend towards increasedpressure, resistance, blood flow, and inside diameter of the portal vein, while shear stress gradually decreased. This led to long-term low shear stress in the portal system. It can be speculated that portal vein remodeling was closely associated with low shear stress. Hence, the biomechanical balance of the venous walls was destroyed, subsequently leading to wall thickening and decreasing compliance. The venous wall structure showed a tendency towards arterialization. These changes caused mechanical unbalance and undoubtedly increased portal hypertension. On the other hand, according to the stress-strain law [16], the changes in structure and composition of the vessels led to alterations in shear stress. This could then maintain the high pressure in the portal system.

Shear stress does not only have an influence on the structure of ECs but also control their gene expression [17]. ECs under stress conditions produce many vasoactive substances, among which NO and NOS are highly relevant to portal hypertension [18]. Some studies have confirmed that low shear stress can induce nitric oxide (NO) production, the endothelium-produced vascular relaxing factor in the peripheral blood of portal hypertension patients and animals [19, 20]. NO and NOS are participating in the transduction of multiple physiological signals. This induces ECs that react and induce a normal level of shear stress by increasing the diameter of the vessel [21]. Damage to ECs decreases the sensitivity of norepinephrine (which is controlled by the ECs) and the release of dilatation factors from the ECs benefits the maintenance of phlebeurysm [22]. ET-1, the most potent vasoconstrictor currently known, strongly promotes the proliferation of ECs and vascular SMCs. Under normal conditions, NO and ET-1 are kept in a relatively balanced state to maintain the mechanical equilibrium of the vessel wall and smooth flow of blood [23]. We found that, during the process of portal hypertension, plasma levels of NO, NOS, and ET-1 gradually increased in direct correlation with changes in the haemodynamic parameters. On one hand, collateral circulation of the portal vein and activation of endogenous or exogenous apoptotic enzymes can result in excessive NO generation [23]. On the other hand, excessive NO generation, secretion of endotoxin, and tumor necrosis factor and increased ET-1 synthesis will lead to smooth muscle cell (SMC) proliferation and hypertrophy, inducing vascular remodeling. In our study SMC proliferation and hypertrophy in the portal vein were observed using optical and electron microscopy. This indirectly proved that NO and ET-1 were closely related with portal venous vascular remodeling.

In the CCl4 group, the differences of blood flow in the portal vein at weeks 6 and 10 were not significantly different. This phenomenon is consistent with the pathogenesis of portal hypertension by “hyperdynamic circulation.” Increasing the vascular relaxing factors NO and NOS led to increasing splanchnic blood flow. Besides a series of compensatory mechanisms, such as increasing PVR and widening portal venous ID, the blood flow in the portal vein did not obviously increase after week 8. Blood staying in the portal vein system for a longer duration induced the formation of a collateral circulation. During the development of portal hypertension, vasodilator and vasoconstrictor were significantly increased in portal blood and might have been associated with the coexistence of high pressure and high blood flow. A continuous high flow led to an increase in ID and OD—a negative feedback regulator of blood flow.

In conclusion, we showed that venous remodeling in the portal vein was always accompanied by the reduction of shear stress. The mechanism by which shear stress controls portal vein remodeling (e.g., mechanical receptor and signal transmission) is still unknown. There are still difficulties in controlling the influence of shear stress on human vascular remodeling, as multiple factors are involved. This needs to be the focus of further research.

## Figures and Tables

**Table 1:** Blood pressure, blood flow and resistance in the portal vein (*χ*  ±  SD).

Group	Number of rats (*n*)	PVP (mmHg)	PVF (ml/min^−1^·kg^−1^)	PVR (mmHg·ml·min^−1^·kg^−1^)
Control	8	4.88 ± 0.29	48.73 ± 2.38	0.090 ± 0.005
Week 4	8	5.27 ± 0.28^★^	52.27 ± 2.69^★^	0.096 ± 0.004^★^
Week 6	8	6.10 ± 0.26^△^	56.66 ± 1.86^△^	0.108 ± 0.004^△^
Week 8	8	6.92 ± 0.32^△^	59.51 ± 2.70^△^	0.120 ± 0.005^△^
Week10	8	7.13 ± 0.33^△^	57.13 ± 2.03^△■^	0.129 ± 0.004^△^

PVF, portal vein flow; PVR, portal vein resistance; PVP, portal venous pressure.

^★^ *P* < 0.05 vs. control group; ^△^ *P* < 0.01 vs. control group; ^■^ *P* > 0.05 vs. week 6.

**Table 2:** Geometric changes in the portal vein (*χ*  ±  SD).

Group	Number of rats (*N*)	ID (×10^2^ *μ*m)	OD (×10^2^ *μ*m)	WT (×10^2^ *μ*m)	CSA (×10^4^ *μ*m^2^)
Control	8	8.08 ± 0.17	10.07 ± 0.14	1.26 ± 0.04	39.42 ± 2.24
Week 4	8	8.24 ± 0.21	10.23 ± 0.14	1.31 ± 0.04	40.52 ± 2.16
Week 6	8	8.68 ± 0.22^△^	11.13 ± 0.21^△^	1.39 ± 0.02^△^	44.52 ± 4.06^△^
Week 8	8	9.03 ± 0.14^△^	12.04 ± 0.13^△^	1.48 ± 0.02^△^	49.92 ± 2.57^△^
Week 10	8	9.24 ± 0.24^△^	12.42 ± 0.25^△^	1.57 ± 0.01^△^	54.52 ± 3.08^△^

CSA, cross-sectional area; ID, internal diameter; OD, outside diameter; WT, wall thickness ^△^ *P* < 0.01 vs. control group.
